# The role of indoleamine 2,3-dioxygenase in LP-BPM5 murine retroviral disease progression

**DOI:** 10.1186/1743-422X-10-154

**Published:** 2013-05-17

**Authors:** Megan A O’Connor, William R Green

**Affiliations:** 1 Department of Microbiology and Immunology, Geisel School of Medicine at Dartmouth, Lebanon, New Hampshire 03756, USA; 2 Norris Cotton Cancer Center, Geisel School of Medicine at Dartmouth, Lebanon, New Hampshire 03756, USA

**Keywords:** LP-BM5 murine retrovirus, Indoleamine 2,3-dioxygenase, Murine AIDS

## Abstract

**Background:**

Indoleamine 2,3-dioxygenase (IDO) is an immunomodulatory intracellular enzyme involved in tryptophan degradation. IDO is induced during cancer and microbial infections by cytokines, ligation of co-stimulatory molecules and/or activation of pattern recognition receptors, ultimately leading to modulation of the immune response. LP-BM5 murine retroviral infection induces murine AIDS (MAIDS), which is characterized by profound and broad immunosuppression of T- and B-cell responses. Our lab has previously described multiple mechanisms regulating the development of immunodeficiency of LP-BM5-induced disease, including Programmed Death 1 (PD-1), IL-10, and T-regulatory (Treg) cells. Immunosuppressive roles of IDO have been demonstrated in other retroviral models, suggesting a possible role for IDO during LP-BM5-induced retroviral disease progression and/or development of viral load.

**Methods:**

Mice deficient in IDO (B6.IDO−/−) and wildtype C57BL/6 (B6) mice were infected with LP-BM5 murine retrovirus. MAIDS and LP-BM5 viral load were assessed at termination.

**Results:**

As expected, IDO was un-inducible in B6.IDO−/− during LP-BM5 infection. B6.IDO−/− mice infected with LP-BM5 retrovirus succumbed to MAIDS as indicated by splenomegaly, serum hyper IgG2a and IgM, decreased responsiveness to B- and T-cell mitogens, conversion of a proportion of CD4^+^ T cells from Thy1.2^+^ to Thy1.2^-^, and increased percentages of CD11b^+^Gr-1^+^ cells. LP-BM5 infected B6.IDO−/− mice also demonstrated the development of roughly equivalent disease kinetics as compared to infected B6 mice. Splenic viral loads of B6 and B6.IDO−/− mice were also equivalent after infection as measured by LP-BM5-specific Def Gag and Eco Gag viral mRNA, determined by qRT-PCR.

**Conclusions:**

Collectively, these results demonstrate IDO neither plays an essential role, nor is required, in LP-BM5-induced disease progression or LP-BM5 viral load.

## Background

Indoleamine 2,3-dioxygenase is an immunomodulatory intracellular enzyme involved in the first, rate-limiting step of tryptophan catabolism [1]. IDO expression is induced by a variety of immune responses, including upregulation of type I (α, β) and type II (γ) interferons (IFNs), and following toll-like receptor (TLR) ligation [2-5]. IDO can also be activated upon ligation of B7 co-stimulatory molecules (CD80 and CD86) on dendritic cells (DCs) by CD28 or CTLA-4 on T-cells [1,3,5-7]. Upon induction, high levels of IDO expression are primarily found in a specialized subset of murine plasmacytoid dendritic cells (pDCs) expressing CD8α, CD19, and B220, but can also be found in other cell types including macrophages, epithelial cells, and some fibroblast-like cells [7-10].

IDO expression induces tryptophan catabolism, resulting in reduced tryptophan levels and increased levels of toxic downstream metabolites (kynurenine, quinolic acid, and picolinic acid), all of which can contribute to T-cell suppression [8,11,12]. T-helper 1 (Th1) cell clones are more sensitive to changes in tryptophan levels than T-helper 2 (Th2) cell clones, resulting in increased Th1-directed immunosuppression [7]. Furthermore, IDO induction is associated with altering the balance of T-cell subsets, increasing the proportion of T-regulatory (Treg) cells and decreasing T-helper 17 (Th17) cells [2,13,14]. Overall, IDO induction causes immunosuppression by dampening Th1 and Th17 cellular responses and enhancing regulatory T-cell responses.

The immunosuppressive role of IDO in modulating immune responses to cancer is well recognized, but the role of IDO during viral infections is less well established [15]. In microbial infections, IDO can act as either an immune suppressor or as an anti-microbial agent, depending on the nature of the infection. IDO-linked immunosuppression occurs during *in vitro* Puumala hantavirus infection [16] and *in vivo* during *Plasmodium* species [17] and *Leishmania major* infections [18]. In contrast, IDO induction acts *in vitro* and/or *in vivo* against bacteria, such as *Chlamydiae*, *Streptococci* and *Staphylococci* species [19-21]; parasites, for example *Toxoplasma gondii*[21]; and viruses, including herpes simplex-2 virus (HSV-2), cytomegalovirus (CMV), hepatitis B virus (HBV), and vaccinia virus infections [22-25]. Increased IDO expression occurs during human immunodeficiency virus (HIV) [26-29], simian immunodeficiency virus (SIV) [13,30] and murine LP-BM5 immunodeficiency-causing retroviral infections [31], yet a comprehensive understanding of the effect of IDO on these retroviral infections has not been provided. Increased IDO mRNA levels in HIV patients have been correlated with increased viral loads, and IDO expression decreases upon antiretroviral therapy (ART), suggesting a direct correlation between IDO and HIV virus propagation [27]. Additional studies in HIV/AIDS systems have further suggested that IDO may play an active immunosuppressive role [7,32,33].

LP-BM5 retroviral infection causes a profound and broad immunodeficiency in susceptible mouse strains such as C57BL/6 (B6); this disease is known as murine AIDS (MAIDS). MAIDS is characterized by early polyclonal T- and B-cell activation, splenomegaly, lymphadenopathy, hypergammaglobulinemia, and a subsequent progressive immunodeficiency of T- and B-cell responses [34-37]. As with AIDS patients, this immunodeficiency leads to an increased incidence of B-cell lineage lymphomas and susceptibility to a variety of opportunistic pathogens [34,38]. In resistant BALB/c mice, we have demonstrated a key protective role of Gag-specific CD8^+^ cytotoxic T-lymphocytes (CTLs), which are critical for controlling LP-BM5 infection and protecting against retroviral pathogenesis [38-42]. We have not been able to detect such robustly protective CTLs in the susceptible B6 model, strongly suggesting that this CTL deficit is critical to the disease induction.

In the LP-BM5 retroviral system immunosuppressive mechanisms have been identified which modulate disease progression. The kinetics and degree of MAIDS pathogenesis are increased after LP-BM5 infection of B6 mice deficient for the Programmed Death 1 (PD-1) gene, but disease can be decreased if instead the interruption of PD-1 signaling is confined to the CD8 compartment [43,44]. Elevated splenic anti-inflammatory IL-10 mRNA levels are found in wildtype (w.t.) B6 mice following LP-BM5 infection [45]. Mice deficient of the IL-10 gene are more susceptible to MAIDS than w.t. B6 mice, presumably because, as we have shown for PD-1, IL-10 may well act to control pathogenic CD4^+^ T-cells that drive LP-BM5-induced disease [43]. Our lab has examined the role of CD4^+^ Tregs, and demonstrated that Forkhead box P3 (FoxP3)^+^ CD4^+^ T-cells increase during the first 2 weeks of infection and plateau at about 18 days post infection (dpi) [44]. Upon initial depletion of natural Tregs, via adoptive transfer of FoxP3^-^CD4^+^ T cells into B6.TCRαR−/− mice, which lack T cells, there was no substantial change in disease severity [44]. However, MAIDS pathogenesis can be reduced upon depletion of FoxP3^+^CD4^+^ Treg cells, in combination with elimination of PD-1 expression on CD8^+^ T-cells [44]. This reduction in the degree of MAIDS seen was apparently due to the observed expansion of a protective CD8^+^ CTL population, which had previously been suppressed by FoxP3^+^ Tregs and/or PD-1 following the infection of intact, un-manipulated B6 mice. These studies further suggest an important role of immunosuppressive mechanisms in either the promotion of, or protection from, LP-BM5-induced pathogenesis.

We have also identified an immunosuppressive monocytic myeloid-derived suppressor cell (MDSC) population (Ly6G^-/low^Ly6C^+^CD11b^+^) [46]. This MDSC population increases during LP-BM5 infection and is capable of suppressing both T- and B-cell responses, in significant part via an inducible nitric oxide synthase (iNOS)-dependent manner [46]. It is possible that the immunosuppressive mechanism(s) of MDSCs in B6 mice could be regulated during LP-BM5 infection, due to the known cross-talk between the iNOS pathway and tryptophan catabolism in other systems [9,11]. Downstream metabolites of tryptophan catabolism can alter iNOS expression; 3-hydroxyanthranilic acid (3HAA) is able to inhibit the activity and expression of iNOS; while picolinic acid is able to induce iNOS in conjunction with IFNγ activation [47,48]. In short, it is becoming appreciated that immunoregulatory mechanisms affect LP-BM5-induced disease progression, suggesting that immunosuppressive roles of IDO could also be playing an important role in MAIDS pathogenesis.

We assessed the role of IDO inhibition on MAIDS progression and LP-BM5 viral load. Compared to other immunodeficiency-causing retroviral infections in primate species, the LP-BM5 murine retroviral system afforded the opportunity for a more comprehensive assessment of IDO, including the use of B6.IDO−/− mice. Our studies suggest a non-essential role for IDO in LP-BM5-induced disease, with little, if any, effect on retroviral load.

## Results and Discussion

### IDO is undetectable in B6.IDO−/− mice following LP-BM5 infection

**Figure 1:**
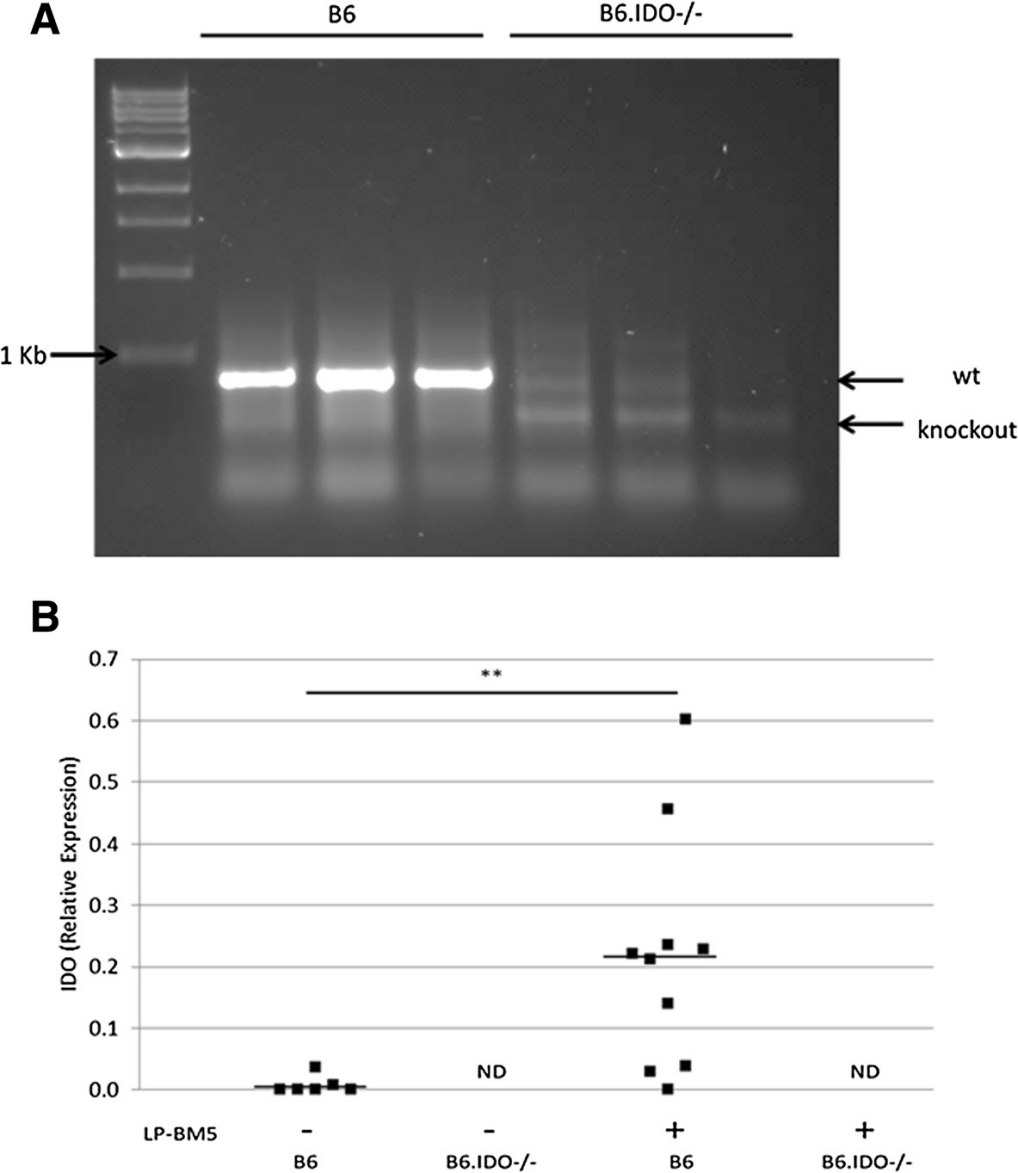
**IDO is Undetectable in B6.IDO−/− Mice Following LP-BM5 Infection.** (**A**) Genomic DNA was isolated from B6 and B6.IDO−/− mice, and the IDO genotypes were determined using PCR. Expected IDO band lengths: w.t. IDO (427 bp) and knockout IDO (280 bp). (**B**) Relative expression of IDO splenic mRNA by qRT-PCR, as compared to β-actin. Lines represent the mean, n = 10, from three independent experiments. IDO mRNA was not detected (ND) in uninfected or infected B6.IDO−/− mice. **p < 0.01, in comparison to uninfected controls, as determined using one-way ANOVA.

The genotype of B6.IDO−/− mice was evaluated, to confirm the absence of the normal w.t. IDO gene. PCR-amplified products of genomic DNA were run on agarose gels to identify the presence of either w.t. or knockout IDO DNA. The wildtype IDO DNA signature (427 bp) was observed only in B6 mice, and the characteristic knockout IDO DNA fragment (280 bp) was found only in B6.IDO−/− mice (Figure 1A). Next, the possible induction of IDO during LP-BM5 infection was determined. B6 and B6.IDO−/− mice were infected in parallel with LP-BM5, and splenic mRNA was assessed at 8 weeks post infection (wpi) for the presence of IDO message using qRT-PCR (Figure 1B). As expected, IDO mRNA was undetectable in all uninfected and infected B6.IDO−/− mice. In contrast, minimal levels of IDO mRNA were detected in uninfected w.t. B6 mice and significantly increased by 8 wpi (Figure 1B). Plasmacytoid dendritic cells (pDCS) have been shown to be major producers of IDO [8], but in our preliminary experiments, no overall obvious difference was seen in the percentage of the pDC cell population at 8 wpi in B6 and B6.IDO−/− mice (data not shown). The induction of IDO in B6 mice after LP-BM5 infection is consistent with a possible role for IDO in LP-BM5-induced pathogenesis and/or viral load.

### B6.IDO−/− mice succumb to MAIDS after LP-BM5 infection

**Figure 2:**
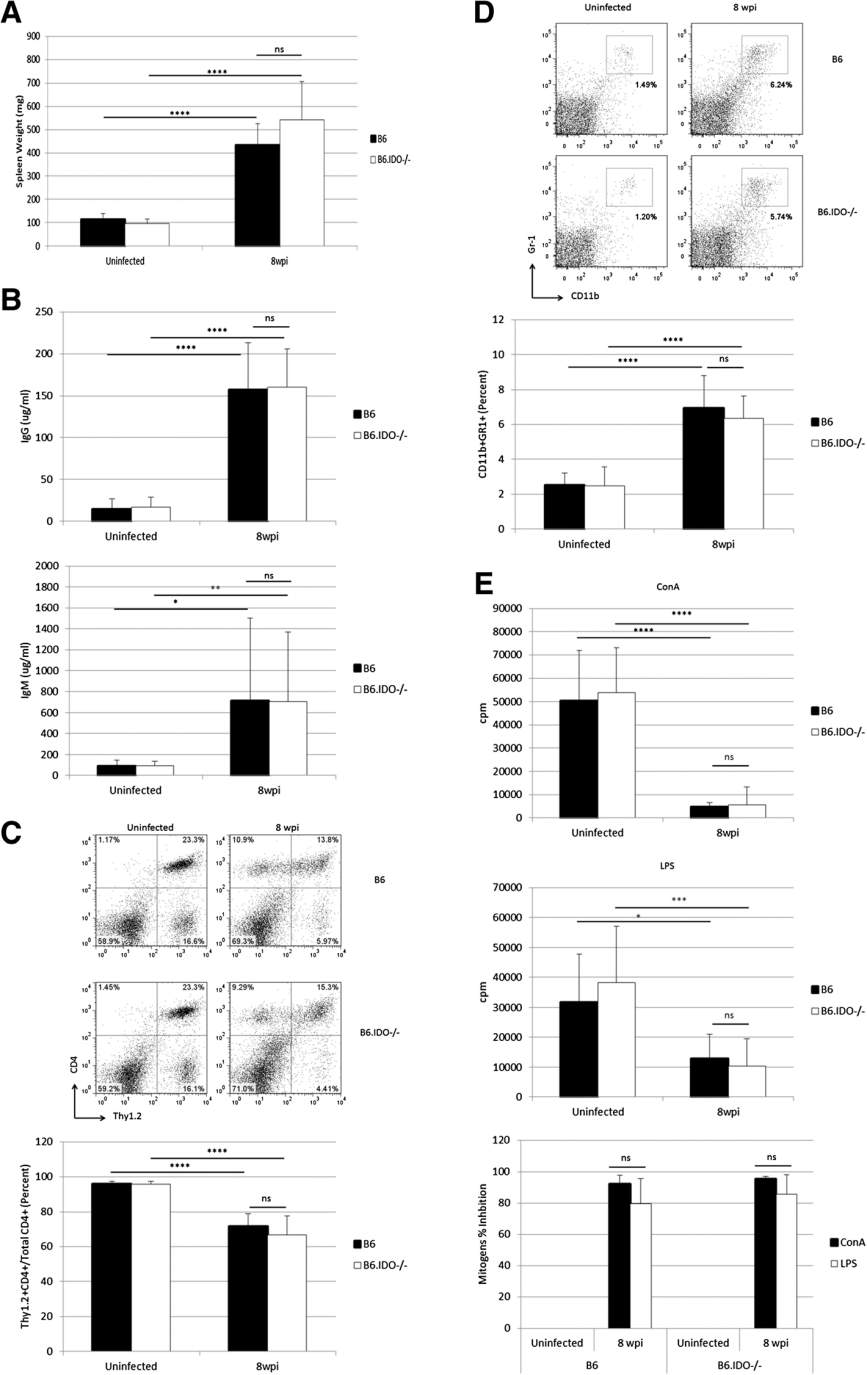
**B6.IDO−/− Mice Succumb to MAIDS After LP-BM5 Infection.** B6 and B6.IDO−/− mice were infected in parallel, with 5x10^4^ pfu of LP-BM5, and MAIDS parameters were assessed at 8 wpi. (**A**) Spleen weights. (**B**) Serum IgG2a and IgM as assessed by ELISA. (**C**) Emergence of Thy1.2^-^CD4^+^ T-cells as assessed by flow cytometry. Percent of Thy1.2^+^CD4^+^ of the total CD4^+^ cell population, calculated from the flow plots of all the experiments. (**D**) Increase in the CD11b^+^GR-1^+^ cell population, assessed by flow cytometry. Percent of CD11b^+^GR-1^+^ of total splenocyte population, calculated from the flow plots of all the experiments. (**E**) Thymidine incorporation after stimulation with ConA and LPS, counts per minute (cpm). Mitogen percent inhibition of the uninfected control to ConA and LPS responses is shown. All histograms represent the mean of samples from four independent experiments, with indicated standard deviations. *p < 0.05; **p < 0.01, ***p < 0.001; **** p < 0.0001, in comparison to uninfected controls, as determined using one-way ANOVA.

To assess the role of IDO in modulating LP-BM5-induced disease, B6 and B6.IDO−/− mice were infected, in parallel, with LP-BM5 and evaluated at 8 wpi for standard MAIDS disease read-outs. Severe splenomegaly occurred in infected B6 and B6.IDO−/− mice at 8 wpi (Figure 2A), the standard time-point for assessment of robust disease [43,49]. As another parameter of the activational aspects of LP-BM5-induced pathogenesis, sera were assessed for IgG2a and IgM levels by ELISA (Figure 2B). MAIDS-associated hypergammaglobulinemia was observed at 8 wpi in both B6 and B6.IDO−/− mice, for IgG2a and IgM. During infection of B6 mice with LP-BM5, there is also the emergence of splenic CD4^+^ T-cells that do not express the characteristic Thy1.2 marker [34,50,51]. A significant decrease in the percentage of Thy1.2^+^CD4^+^ cells of the total CD4^+^ population was observed in w.t. B6, as expected, but also in B6.IDO−/− mice by 8 wpi (Figure 2C). As another indicator of LP-BM5 pathogenesis during B6 infection, our lab has identified an increase in a CD11b^+^Gr-1^+^ cell population, as early as 5 wpi [46]. Here, we further demonstrate B6.IDO−/− mice also have a significant increase in the CD11b^+^Gr-1^+^ cell population at 8 wpi (Figure 2D). Because it is a monocytic MDSC subpopulation (Ly6G^-/low^Ly6C^+^CD11b^+^) from LP-BM5 infected B6 mice that is capable of suppressing T- and B-cell responses, in part via an iNOS-dependent manner [46], we also demonstrated here that LP-BM5 infection-dependent increases in the Ly6G^-/low^Ly6C^+^CD11b^+^ population are readily detectible in B6.IDO−/−, as well as B6, mice (data not shown). Furthermore, in preliminary experiments, Ly6G^-^CD11b^+^ MDSCs derived from infected B6.IDO−/− mice have roughly similar suppressive capabilities against the response to polyclonal LPS stimulation in comparison to MDSCs derived from infected B6 mice (data not shown).

Lastly, immunosuppression was measured, as demonstrated by the standard MAIDS read-out of T- and B-cell responsiveness to polyclonal mitogen activation [51]. Isolated splenocytes from uninfected and infected mice were stimulated with ConA or LPS, and proliferation was assessed 72 hours post stimulation. At 8 wpi, B6 mice have a significantly decreased responsiveness to ConA and LPS (Figure 2E). B6.IDO−/− mice also exhibit significant and comparable levels of immunosuppression in response to these T- and B-cell mitogens, as compared to uninfected controls (Figure 2E).

Based on the collective assessment of all MAIDS parameters, B6.IDO−/− mice are susceptible to MAIDS and experience immunodeficiency with similar severity to B6 mice.

### IDO is not required for MAIDS at different timepoints or doses of LP-BM5

**Figure 3:**
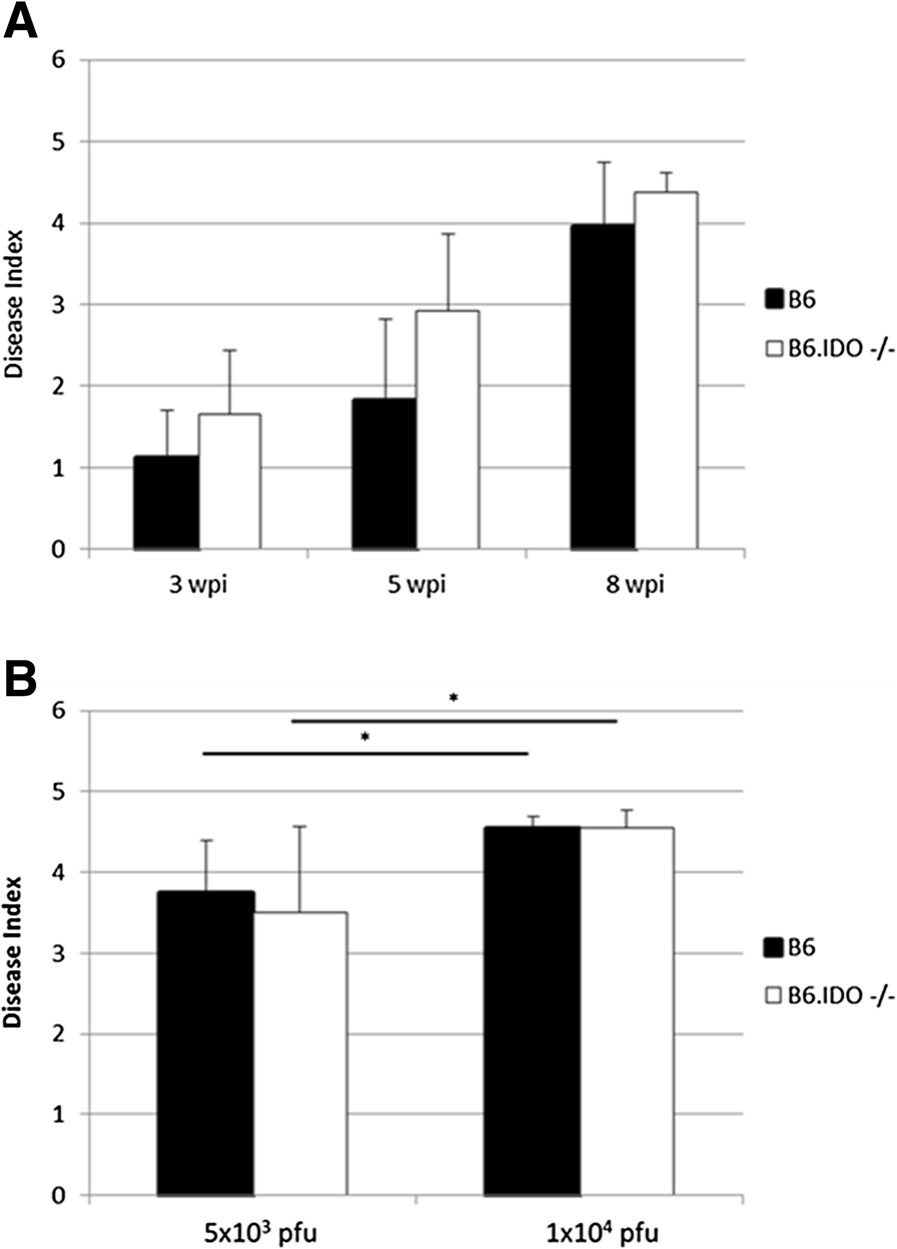
**IDO is Not Required for MAIDS at Different Timepoints or Doses of LP-BM5.** LP-BM5-induced disease kinetics were assessed in B6 and B6.IDO−/− mice. (**A**) B6 and B6.IDO−/− mice were infected in parallel, with 5x10^4^ pfu of LP-BM5 virus. MAIDS parameters were assessed at 3, 5, and 8 wpi and disease index was determined. (**B**) B6 and B6.IDO−/− mice were infected with LP-BM5 at a dose of 5x10^3^ or 1x10^4^ pfu. Disease parameters were assessed at 8 wpi and disease index was calculated. Histograms represent the mean, (n = 3), with indicated standard deviations. The patterns of the presented results are representative of two additional experiments, which provided a similar pattern of results. *p < 0.05, in comparison to 5x10^3^ pfu, as determined using one-way ANOVA.

Although IDO thus appears to have little overall effect on MAIDS at 8 wpi, the kinetics of disease progression may differ for B6 and B6.IDO−/− mice. Timepoints earlier than the standard 8 wpi endpoint determination were therefore assessed. In these experiments, to better compare overall disease levels, mice were first evaluated for standard MAIDS parameters, including splenomegaly, hypergammaglobulinemia, responsiveness to T- and B-cell mitogens, and emergence of Thy1.2^-^CD4^+^ T-cells, as described in Figure 2. Then disease index, as previously described, was calculated, ranging from 0 (no disease) to 5 (severe disease) [41]. At the standard dose of 5×10^4^ pfu of LP-BM5, disease kinetics between B6 and B6.IDO−/− mice were generally similar, with little disease at 3 wpi and a gradual increase in disease over the course of infection as assessed at 5 and 8 wpi (Figure 3A). No significant difference in the extent of disease was demonstrated between the infected groups at any of the time points.

It was next determined if B6 and B6.IDO−/− mice have different susceptibilities to disease after infection by lower viral doses. B6 and B6.IDO−/− mice were infected in parallel with either 5×10^3^ or 1×10^4^ pfu of LP-BM5, and disease index was assessed at 8 wpi (Figure 3B). Infected B6 and B6.IDO−/− mice succumbed to substantial, and roughly equivalent, MAIDS at these two lower viral inoculums, with no observed significant differences in the quantification of disease. Thus, B6.IDO−/− mice are generally as susceptible to MAIDS at multiple viral doses as compared to prototypic susceptible B6 mice.

### Infected B6.IDO−/− mice do not display an altered LP-BM5 viral load

**Figure 4:**
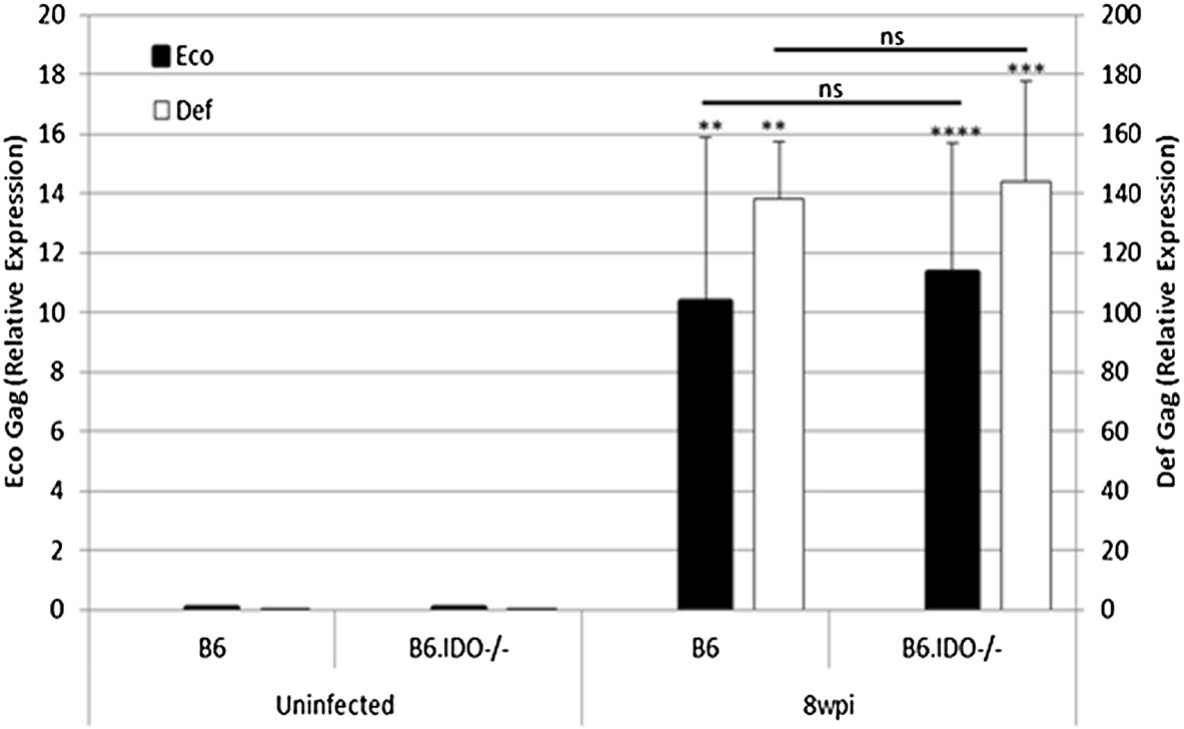
**Infected B6.IDO−/− Mice Do Not Display An Altered LP-BM5 Viral Load.** B6 and B6.IDO−/− mice were infected in parallel, with 5x10^4^ pfu, and splenic mRNA was extracted at 8 wpi. LP-BM5 Eco Gag and Def Gag mRNA were assessed by qRT-PCR, relative to β-actin [63]. Histograms represent the means, (n = 4), with indicated standard deviations. The patterns of the presented results are representative of three additional experiments, which provided a similar pattern of results. **p < 0.01; ***p < 0.001; ****p < 0.0001, in comparison to uninfected controls, as determined using one-way ANOVA.

While no apparent differences were observed between B6 and B6.IDO−/− mice in regards to susceptibility to MAIDS progression, there may be differences in viral load. For example, in a w.t. LP-BM5 infected B6 mouse, IDO may not suppress the pathogenic CD4^+^ T cells that are necessary for disease initiation and progression [51], but rather IDO may have an effect on viral spread and subsequent viral load. To address this question, splenic mRNA was extracted from uninfected and infected B6 and B6.IDO−/− mice. Splenic mRNA was assessed for LP-BM5-associated ecotropic (Eco) helper and defective (Def) Gag viral sequences. Compared to essentially no detectable signal in uninfected mice, a significant increase in Eco Gag and Def Gag mRNA was detected in B6 and B6.IDO−/− mice (Figure 4). Furthermore, no significant difference was observed between either Eco Gag or Def Gag of the infected B6.IDO−/− mice in comparison to infected B6 mice. Thus, we could not detect differences in accumulated retroviral load, suggesting that LP-BM5 viral replication and spread were not substantially altered in B6.IDO−/− mice.

In summary, we demonstrated that B6.IDO−/− mice were susceptible to LP-BM5 induced disease and had relatively equivalent levels of disease as compared to infected B6 mice, at multiple timepoints and at varying doses. We further demonstrated that viral load was essentially unaltered in infected B6.IDO−/− mice in comparison to infected B6 mice. Our results are interesting to compare to those of *Hoshi, et al*., who recently reported an increase in IDO mRNA, protein levels, and enzymatic activity in response to LP-BM5 infection [31]. Their results are consistent with our results herein, as we demonstrated an increase in splenic IDO mRNA at 8 wpi (Figure 1A). However, it remains unclear whether viral infection per se and/or the host immune response is responsible for the induction of IDO. In any event, regardless of the source(s) of the induced IDO, this response does not appear, in itself, to be crucial in the balance of host resistance factors versus retroviral pathogenesis mechanisms that lead to the profound disease observed in LP-BM5 infected, susceptible B6 mice.

*Hoshi, et al.* also infected B6.IDO−/− mice or treated infected B6 mice with 1-methyl tryptophan (1MT), an IDO inhibitor, and demonstrated an increased responsiveness to the T-cell mitogen ConA, in comparison to infected B6 mice, suggesting decreased immunosuppression when IDO activity was not present [31]. However, no uninfected control mice were reported for comparison, thus making it unclear what percentage of mitogen responsiveness was restored in IDO deficient mice. In contrast, our data demonstrate that B6.IDO−/− mice exhibited substantially decreased responsiveness to both T-cell (ConA) and B-cell (LPS) mitogens in comparison to stimulated uninfected controls, and there was no difference in the extent of immunodeficiency as compared to infected w.t. mice (Figure 2E).

Due to these differential results in LP-BM5-induced immunosuppression between our data and those of *Hoshi, et al.*, we employed a more comprehensive, yet accepted, panel of MAIDS parameters, to obtain a broader understanding of the role of IDO in LP-BM5-induced disease. All MAIDS parameters examined herein indicated LP-BM5-induced pathogenesis in infected B6.IDO−/− at roughly equivalent levels to those of infected B6 mice (Figures 2 and 3). *Hoshi, et al.* also reported decreased splenic viral copies of Def Gag at 8 wpi in either infected B6.IDO−/− mice or infected B6 mice treated with 1MT [31]. In our studies, we found no significant difference in splenic mRNA for Def Gag and Eco Gag at any of the tested timepoints (3, 5, and 8 wpi) (Figure 4 and data not shown). Although our LP-BM5 inoculum and that used by *Hoshi, et al.* appear to be comparable doses, we wanted to confirm that the effects seen were not due to our administration of a larger viral dose. To assess this, we compared three infectious viral doses, including two that were lower than our standard inoculum: 5x10^4^, 1x10^4^, and 5x10^3^ pfu. However, at each dose essentially no difference in splenic mRNA for Def or Eco Gag was observed between infected B6 and B6.IDO−/− mice (data not shown), consistent with our finding of no differential levels of disease (Figure 3B). Alternatively, variance in the proportion of the defective and ecotropic genomes within the different LP-BM5 viral preparations might explain the differences seen between the two studies. Whether this potential variable of the pathogenic LP-BM5 Def Gag content or distribution among virions is responsible for the different results obtained here, versus by *Hoshi, et al.*, or whether other factors are also responsible, is unclear. Future studies to clarify the differences may be informative for a better understanding of the regulation of LP-BM5-induced pathogenesis. In conclusion, our data clearly demonstrate a non-essential role of IDO in MAIDS or viral load development following LP-BM5 infection of susceptible B6 mice.

It is also instructive to consider the effects of IDO, or its absence, in other viral systems. IDO has been shown to have differential effects during HSV-1 infection. *In vitro* studies have demonstrated that IDO can exert an anti-viral effect against HSV-1 infection, which appears to be mediated in part by IFNγ and/or IL-1 [52,53]. It was further demonstrated *in vivo* that mice infected with HSV-1, which consequently developed signs of encephalitis, had increased levels of quinolic acid, a downstream metabolite of tryptophan catabolism [54]. These studies suggest an anti-viral role for IDO during HSV-1 infection. However, treatment of mice with 1MT during HSV-1 infection had no detectable effect on HSV-1 replication or on the survival of the infected mice [55]. Thus, IDO may act as an immunosuppressive molecule during viral infections depending on the model of infection (*in vitro* versus *in vivo*), and, most likely, also by the presence/absence of other immunomodulatory signals within the micro-environment.

With respect to other immunodeficiency-causing retroviruses, IDO has been implicated as a potential therapeutic target against HIV/AIDS, and the use of IDO inhibitors has recently been explored within SIV models, but with mixed results. *In vitro* studies have shown improved proliferative responses of CD4^+^ and CD8^+^ T-cells from immunosuppressed HIV-infected patients by addition of 1MT [27,33,56]. There is also *in vitro* evidence that IDO acts differentially to suppress the T-cell response against HIV infection: IDO can arrest CD4^+^ T cells in the G1-S transition phase of the cell cycle, but can suppress CD8^+^ T cells by reduced expression of the CD28 co-stimulatory molecule [56]. In contrast, *in vivo* studies using 1MT, to inhibit IDO, have led to variable results. In a murine model of HIV-induced encephalitis, in which human peripheral-blood lymphocytes (hu-PBLs) are given to nonobese diabetic-severe combined immunodeficient (NOD/SCID) mice and then are infected by injection of HIV-1-infected monocyte-derived macrophages, treatment with 1MT lead to an increase in the HIV-specific CTL response and essentially elimination of HIV-infected macrophages from the brain [33]. Similarly, SIV-infected rhesus macaques that were treated with 1MT, and also on antiretroviral treatment (ART), displayed reduced SIV RNA levels in the spleen and plasma [57]. In this same study, however, macaques not on ART and treated with 1MT exhibited no change in viral levels [57]. Likewise, ART-treated macaques also given 1MT therapy demonstrated transiently increased plasma levels of tryptophan, but no reduction in kynurenine [57]. In a seemingly similar study, different results were obtained: SIV-infected ART-treated macaques also given 1MT demonstrated no effect on tryptophan catabolism or on viral load [58]. Clearly, more work still needs to be done to fully understand the potential use of 1MT or other agents that target IDO, or the pathways involving IDO, before its potential to treat HIV/AIDS patients can be determined. Therefore, the study of well-controlled animal models of retrovirus-induced immunodeficiency will undoubtedly be important to sort out these complex interactions and sometimes conflicting initial results.

## Conclusions

Our data demonstrate a non-essential role of IDO in MAIDS or viral load following LP-BM5 infection of susceptible C57BL/6 mice.

## Methods

### Mice

C57BL/6 (B6) mice were purchased from the National Cancer Institute (NCI, Bethesda, MD) and housed in the Center for Comparative Medicine and Research (CCMR) at the Geisel School of Medicine at Dartmouth. Breeding pairs of B6.IDO−/− mice were purchased from The Jackson Laboratory (Ban Harbor, ME) and bred in-house at the CCMR. All animal experiments were done with the approval of the Institutional Animal Care and Use Committee of Dartmouth College, and in conjunction with the Dartmouth Center for Comparative Medicine and Research, an AALAC approved animal facility. This institution has an Animal Welfare Assurance on file (A3259-01). The genetic backgrounds of B6.IDO−/− mice were assessed at the DartMouse™ Speed Congenic Core Facility at the Geisel School of Medicine at Dartmouth, which uses the Illumina, Inc. (San Diego, CA) GoldenGate Genotyping Assay to interrogate 1449 SNPs spread throughout the genome. The raw SNP data were analyzed using DartMouse’s SNaP-Map™ and Map-Synth™ software, allowing the determination for each mouse of the genetic background at each SNP location.

### LP-BM5 virus inoculation

LP-BM5 retrovirus was prepared as previously described [59,60]. All mice were given intraperitoneal injections between 6 and 8 weeks of age with one of the following doses: 5×10^4^ pfu, 1×10^4^ pfu, or 5×10^3^ pfu of LP-BM5.

### Genotyping PCR

Tail snips were taken and digested with 25 mM NaOH and 0.2 mM EDTA for 1 hour at 98°C. 40 mM TrisHCl (pH5.5) was added to samples and supernatant was isolated after centrifugation. Genomic DNA was amplified by PCR using primers for both w.t. IDO and knockout IDO from The Jackson Laboratory website: w.t. IDO Primer 1: 5’-TGG AGC TGC CCG ACG C-3’, w.t. IDO Primer 2: 5’-TAC CTT CCG AGC CCA GAC AC-3’, knockout IDO Primer 1: 5’-CTT GGG TGG AGA GGC TAT TC-3’, knockout IDO Primer 2: 5’-AGG TGA GAT GAC AGG AGA TC-3’. PCR products were run on a 1.5% agarose gel and visualized.

### Splenocyte responses to mitogens

Splenocytes were isolated as previously described and responses to Concanavalin A (ConA) and lipopolysaccharide (LPS) mitogens were measured [51]. Briefly, splenocytes were plated in triplicate in a flat-bottom 96-well plate and stimulated with a final concentration in the well of 0.75ug/ml of ConA, 10ug/ml of LPS or supplemented media alone. After 72 hours, cells were pulsed with 1uCi of [^3^H] thymidine (Dupont NEN, Boston, MA) and harvested 6 hrs later for assessment of thymidine incorporation.

### ELISA determination of serum Ig

Serum hyper IgG2a and IgM were assessed by an enzyme-linked immunosorbent assay (ELISA) as detailed previously [61,62]. Affinity-purified goat anti-mouse IgG2a or IgM antibodies were used to coat 96-well plates (Southern Biotechnology Associates, Birmingham, AL) and the ELISA was developed using phosphate substrate (p4744, Sigma-Aldrich).

### Flow cytometry

Surface staining of splenocytes was performed as previously described [51]. Cells were stained with FITC-, PE-, PerCP- and APC- conjugated fluorescent antibodies and the resulting fluorescence was measured on FACSCalibur or FACSCanto Instruments (BD Biosciences) to detect murine CD4 (RM4-5), Thy1.2 (53–2.1), CD11b (M1/70), and Gr-1 (RB6-8C5) (BioLegend and BD Biosciences). Appropriate amounts of FITC-, PE-, PerCP- and APC- conjugated Ig isotypes were used as controls. Data were analyzed using FlowJo software (Tree Star, Inc.).

### RNA isolation and real-time quantitative PCR

Total RNA was isolated from the spleen, for quantitative RT-PCR using RNeasy columns, including DNAse-I treatment (Qiagen, Valencia, CA). DNAse-free RNA was reverse transcribed using the iScript cDNA synthesis kit (Qiagen, Valencia, CA). qRT-PCR was performed using SYBR Green PCR Core kit (Applied Biosystems, Foster City, CA) on an iCyler iQ instrument (Bio-Rad, Hercules, CA). Relative expression of Eco Gag, Def Gag, and IDO were evaluated in comparison to β-actin controls as previously described [63]. IDO primers were forward 5’-AGA CCA CCA CAT AGA TGA AG-3’ and reverse 5’-CCA CCA ATA GAG AGA CGA GGA-3’ as previously described [5].

### Calculation of disease index

The disease index was calculated as previously described [41]. Briefly, the percent disease was calculated for each mouse after measurement of each of the standard panel of MAIDS disease parameters (splenomegaly, serum levels of IgG2a and IgM, responsiveness to ConA and LPS, and percent of Thy1.2^+^CD4^+^ T-cells). Disease index values were designated based on the percentages of disease for each parameter. Mice with less than 1% disease were considered negative for disease and given a value of 0, mice with 1-20% disease were assigned a value of 0.5, mice with 21-40% disease were given a value of 1, mice with 41-60% disease were assigned a value of 2, mice with 61-80% disease were given a value of 3, mice with 81-100% disease were assigned a value of 4, and mice with disease greater than 100% were given a value of 5.

## Abbreviations

IDO: Indoleamine 2,3-dioxygenase; MAIDS: Murine AIDS; HIV: Human immunodeficiency virus; SIV: Simian immunodeficiency virus.

## Competing interests

The authors declare that they have no competing interests.

## Authors’ contributions

MAO and WRG conceived of the study and contributed to the design of the study. MAO performed all experiments, analyzed the data, and wrote the manuscript. Both authors read and approved the final manuscript.
